# Exploring repellency of odors from non-host plants native to Xinjiang, China to *Aphis gossypii*

**DOI:** 10.3389/fpls.2025.1563752

**Published:** 2025-05-28

**Authors:** Zhipeng Bian, Ying Zhang, Bing Liu, Tao Zhang, Haibin Yuan, Yanhui Lu

**Affiliations:** ^1^ Department of Plant Protection, Jilin Agricultural University, Changchun, China; ^2^ State Key Laboratory for Biology of Plant Diseases and Insect Pests, Institute of Plant Protection, Chinese Academy of Agricultural Sciences, Beijing, China; ^3^ State Key Laboratory of IPM on Crops in Northern Region of North China, Institute of Plant Protection, Hebei Academy of Agriculture and Forestry Sciences, Baoding, China

**Keywords:** *Aphis gossypii*, gas chromatography-mass spectrometry (GC-MS), electroantennogram (EAG), volatile compounds, repellent effects

## Abstract

**Introduction:**

The cotton aphid (*Aphis gossypii* Glover) is a major global agricultural pest that damages cotton and numerous other economically significant crops through feeding and virus transmission. It possesses high adaptability and rapid reproduction rates, contributing to widespread resistance to chemical insecticides and thereby reducing the effectiveness of such control methods. It should be noted that plants have developed advanced chemical defense mechanisms over long periods of synergistic evolution, allowing them to synthesize volatile compounds. These compounds not only defend against herbivorous insects but also crucially reduce the development of pest resistance. Consequently, this study strives to explore plant-emitted volatiles as a potential eco-friendly alternative for aphid management.

**Methods:**

In this study, we first tested the behavioral responses of *A. gossypii* to the volatile blends of fifteen native plant species in Xinjiang, China, using a Y-tube olfactometer and cage experiments. We identified six out of fifteen plant species that were repellent to *A. gossypii*. We then collected the volatile compounds of repellent plants using a headspace collection method and used gas chromatography-mass spectrometry (GC-MS) to identify the key components. The antennal responses of winged *A. gossypii* to the compounds were evaluated with an antennal potential test. Finally, further testing using a Y-tube olfactometer and Petri dish experiments.

**Results:**

We identified six out of fifteen plant species (i.e. *Anethum graveolens* L., *Juglans regia* L., *Rhaponticum repens* L., *Karelinia caspia Pall*., *Launaea polydichotoma Ostenf*., and *Brassica rapa* L.) that were repellent to *A. gossypii*. We collected the volatile compounds of these six repellent plants and identified thirty-one key components. Our electroantennogram (EAG) tests revealed that sixteen of the thirty-one compounds caused significant antennal responses in winged *A. gossypii.* Further testing using a Y-tube olfactometer and Petri dish experiments confirmed fourteen compounds that repelled intact, winged cotton aphids.

**Discussion:**

Our study report that the volatiles of four plant species – *R. repens, K.caspia, L. polydichotoma*, and *B. rapa* – present a significant repellent effect on winged cotton aphids, suggesting that these compounds might be useful for eco-friendly cotton aphid pest management. These results provide essential theoretical foundations and practical knowledge for the application of plantderived repellent volatiles.

## 1 Introduction

The cotton aphid, *Aphis gossypii* (Glover) (Hemiptera: Aphididae), inflicts both direct and indirect damage on cotton and numerous agricultural crops from the families Cucurbitaceae, Rutaceae, and Malvaceae, resulting in significant economic losses worldwide (Somar et al., 2019). Being a piercing-sucking insect, it instigates leaf curling and has the potential to transmit an array of viral diseases. Two morphs – winged and wingless aphids – occur in distinct seasons. Compared to wingless aphids, winged aphids can disperse over considerable distances with the aid of wind, causing damage beyond their original infestation areas. Nevertheless, monitoring and predicting the arrival time and population of winged aphids remains challenging, thereby increasing the threat they pose to agricultural production (Li et al., 2022). Chemical control remains the main strategy for managing *A. gossypii*; however, the sustained use of chemical pesticides has led to significant resistance in *A. gossypii* to various insecticides, including organophosphates (Wang et al., 2007), pyrethroids (Chen et al., 2017), and neonicotinoids (Bass et al., 2015; Li et al., 2016; de Little et al., 2017), thereby complicating long-term pest control efforts. Moreover, the non-selective nature of chemical pesticides can negatively influence beneficial insects, such as natural enemies and pollinators, adding further complications to integrated pest management (Dai et al., 2020). Therefore, alternative control strategies that minimize reliance on chemical pesticides are critically required for the effective management of *A. gossypii*.

Volatile compounds released into the air by plants can effectively repel some pests as natural insect repellents, disrupting their olfactory and behavioral responses. Prior research indicates that the effectiveness of these volatile compounds at repelling pests varies among different plant species (Szendrei and Rodriguez-Saona, 2010).

In nature ecosystems, many plants can repel pests by releasing volatile compounds, and their essential oil could be more effective than the live plants themselves (Dunan et al., 2021). For example, intercropping cotton with *Allium cepa* L. or *Allium sativum* L. can repel *A. gossypii* on cotton plants (Yang et al., 2023); *Lavandula pinnata* L. and *Rosmarinus officinalis* L. can deter *Empoasca vitis* Gothe (Zhang et al., 2014); if *Ocimum basilicum* L. is cultivated adjacent to *Amaranthus hybridus* L., it wards off *Aphis craccivora* Koch (Yarou et al., 2020); and *Foeniculum vulgare* Mill. repels the aphid *Macrosiphum euphorbiae* Thomas. As the environmental impact of pesticides increases, plant-derived pesticides and insect repellents, such as azadirachtin and citronella oil, have become crucial for sustainable pest control (Haritha et al., 2021; Khursheed et al., 2022). Such plant-derived pesticides are environmentally friendly, relatively safe for non-target organisms, and hardly contribute to the development of pest resistance (Cantrell et al., 2012). Therefore, non-host plants play a vital role in conserving biodiversity and have significant potential for developing plant-based insecticides for controlling agricultural pests. Therefore, we can minimize pest damage to crops by using these plant resources wisely.

Xinjiang, located in western China, is known for its diverse geography and complex ecosystems, which include both temperate and warm temperate desert zones (Zhao et al., 2017). The region’s varied ecosystems comprise forests, grasslands, deserts, oases, and wetlands (Xi et al., 2006), providing a robust habitat foundation for species diversity (Taoerdahong et al., 2023). Numerous native plants in Xinjiang hold substantial ecological and economic value for local agricultural production. Recent studies have ascertained that specific native plants have significant biological activity against pests. For example, ethanol extracts of *Datura stramonium* L. and *Sophora alopecuroides* L. have reportedly shown substantial biological activity against *A. gossypii* (Fang, 2021). Similarly, compounds from *Ammopiptanthus nanus* Popov populations in Xinjiang enhance pest resistance through specific signaling pathways (Wang et al., 2024a). Furthermore, many native plants in Xinjiang produce unique volatile odors. These not only serve as distinctive biological traits but may also act as defense mechanisms against pests. For instance, the aromas of *Ocimum basilicum* L. and *Mentha canadensis* L., which are abundant in terpenoids, phenols, and aldehydes, contribute to their individual scents and have proven to effectively repel winged *A. gossypii*. In addition, the odors of functional plants such as *Cnidium monnieri* L. and *Brassica napus* L. can attract predatory natural enemies, thus controlling pests in cotton fields effectively (Peng et al., 2024). Therefore, further investigations into the characteristics of native plants could offer valuable insights for the development of green agriculture and sustainable pest control strategies.

In this study, we examined fifteen native plants from Xinjiang, China (see species list in the Methods section) to identify those that display repellent effects on winged *A. gossypii*, focusing particularly on plants with relatively low aphid populations. The unique volatiles these plants emitted suggested potential repellent properties, which prompted their selection for experimental examination. Through the analysis of the chemical composition of these volatile compounds, we aimed to isolate the active compounds responsible for repelling *A. gossypii*. This provides a theoretical basis for environmentally friendly pest control methods.

## 2 Materials and methods

### 2.1 Insects

Aphids were obtained from a laboratory colony of *A. gossypii* established in 2024 using individuals collected from the experimental cotton fields at Korla Experimental Station in Xinjiang, China (41.45°N, 85.48°E), which belongs to the Institute of Plant Protection, Chinese Academy of Agricultural Sciences (IPP, CAAS). The aphid colony was maintained on cotton (Zhongmian 49) seedlings at the five-leaf stage in a glasshouse under controlled conditions (25 ± 1°C, 16 h:8 h, light:dark photoperiod).

### 2.2 Test plants and reagents

This experiment involved the testing of fifteen plant species, namely *Anethum graveolens* L., *Juglans regia* L., *Rhaponticum repens* L., *Karelinia caspia* Pall., *Launaea polydichotoma* Ostenf., *Brassica rapa* L., *Tamarix ramosissima* Ledeb., *Haloxylon ammodendron* C. A. Mey., *Apocynum venetum* L., *Cynanchum sibiricum* Willd., *Lycium ruthenicum* Murr., *Elaeagnus angustifolia* L., *Nepeta cataria* L., *Neotrinia* sp*lendens* Trin., and *Caragana halodendron* Pall., as detailed in **Table 1**. All plant materials were field-collected and immediately transported to our lab due to the difficulties in maintaining entire plants alive in a laboratory environment. To ensure consistency throughout the experiments, a standardized weight of 50 g per sample was used for all species, regardless of their varied sizes.

**Table 1:** Plant species selected for behavioral bioassays and chemical analyses.

No.	Scientific name	Family	Genus	Plant tissues
1	*Anethum graveolens* L.	Apiaceae	Anethum	Stems, leaves
2	*Karelinia caspia* Pall.	Asteraceae	Karelinia	Stems, leaves
3	*Juglans regia* L.	Juglandaceae	Juglans	Leaves
4	*Launaea polydichotoma* Ostenf.	Asteraceae	Hexinia	Stems, leaves
5	*Tamarix ramosissima* Ledeb.	Tamaricaceae	Tamarix	Stems, leaves
6	*Haloxylon ammodendron* C. A. Mey.	Chenopodiaceae	Haloxylon	Stems, leaves
7	*Apocynum venetum* L.	Apocynaceae	Apocynum	Stems, leaves
8	*Cynanchum sibiricum* Willd.	Asclepiadaceae	Cynanchum	Stems, leaves
9	*Lycium ruthenicum* Murr.	Solanaceae	Lycium	Stems, leaves
10	*Elaeagnus angustifolia* L.	Elaeagnaceae	Elaeagnus	Stems, leaves
11	*Brassica rapa* L.	Brassicaceae	Brassica	Stems, leaves
12	*Nepeta cataria* L.	Lamiaceae	Nepeta	Stems, leaves
13	*Neotrinia* sp*lendens* Trin.	Poaceae	Achnatherum	Stems, leaves
14	*Rhaponticum repens* L.	Asteraceae	Stemmacantha	Stems, leaves
15	*Caragana halodendron* Pall.	Fabaceae	Caragana	Leaves, fruits

In this study, thirty-one compounds identified by gas chromatography-mass spectrometry (GC-MS) were evaluated. All the tested compounds were procured from Aladdin Biochemical Technology Co., Ltd Shanghai, China. Further details can be found in **Supplementary Table S1**.

### 2.3 Screening of fifteen plant species for repellent activity against winged *A. gossypii*

#### 2.3.1 Y-tube olfactometer tests of plant volatiles against winged *A. gossypii*

The behavioral responses of winged *A. gossypii* were evaluated using a Y-tube olfactometer, adhering to the methods delineated by Zhang et al. (2024) with minor modifications. The two arms of the Y-tube olfactometer were each connected to source bottles, one containing 50 g of the plant under test, and the other utilizing air as a blank control. An aphid was delicately placed in the primary arm of the Y-tube olfactometer using a brush. The timing commenced once the aphid crossed the midway point of the primary arm. If the aphid traversed one-third of either arm and remained stationary for longer than 5 s, this was documented as a choice. Conversely, if no decisive choice was observed within 5 min, it was noted as a non-response. Post-testing of every 5 aphids, the positions of the two arms were interchanged, and after every 10 aphids, a new, sterile Y-tube olfactometer was employed. A total of 90 aphids were tested per plant species, with each aphid used only once during the bioassays. After the experiment, the Y-tube olfactometer along with the source bottles and the Teflon tubes interconnecting the arms were cleansed with alcohol and air-dried. Before the onset of testing, to validate any directional bias in the setup, a four-leaf cotton plant was placed in each source bottle. The subsequent experiment was deemed valid only if the number of aphids choosing each arm in the preliminary test was evenly balanced.

#### 2.3.2 Selection by winged aphids of plants in a cage trial

This experiment was conducted using insect cages, each covered with 80-mesh nylon and with dimensions of 80 cm on all sides. The procedure was initiated daily at 9:00 AM. A four-leaf cotton seedling was positioned at two diagonally opposite corners within each cage. Following the placement, all experimental plants were washed with distilled water and left to air dry naturally. Subsequently, branches and leaves weighing 20 g were assembled in a mesh bag and fastened around a cotton seedling. The setup was completed in a greenhouse that ensured uniform lighting, a consistent temperature of 25°C and maintained a relative humidity between 60%–75% as cited in (Anderson et al., 2013). After 24 h, aphids that had flown to and settled on each of the two cotton seedlings were counted. This procedure was repeated thrice for each type of odor-source plant.

### 2.4 Collection and identification of volatile compounds from six repellent plant species

We gathered volatiles from each of six potentially repellent native plants (as suggested by previous Y-tube and cage tests) using the headspace collection method following previously cited research with slight modifications (Cai, 2020). Plants (*A. graveolens*, *J. regia*, *R. repens*, *K. caspia*, *L. polydichotoma*, *B. rapa*) exhibiting consistent growth (the pre-flowering stage) were selected for the experiment. The leaves were rinsed with distilled water using a spray bottle to remove dust and then allowed to air dry naturally. Once the plants were completely dry, we weighed 100 g of plant material and securely wrapped the plants above the roots in an oven bag (30 cm × 40 cm), sealing the bottom with tape.

The tubes filled with the adsorbent were sequentially rinsed twice using 1 mL of acetone, ethyl acetate, and n-hexane. The end of the atmospheric sampler’s outlet was connected to the activated carbon sampling tube through a Teflon tube. Airflow was directed through the activated carbon tube and returned to the oven bag’s inlet. A Teflon tube was then attached to the oven bag’s top corner outlet, further linking it to a glass tube containing 200 mg of Porapak Q (80–100 mesh), which served to adsorb any volatiles present. The airflow was subsequently rerouted to the inlet of the atmospheric sampler via the Teflon tube. The system operated at a consistent flow rate of 1 L/min, circulating the air for 4 h. Post collection, the tubes loaded with adsorbent were detached, sealed with sealing film, and desorbed inside a fume hood. The tubes were next rinsed using 1 mL of chromatographic-grade n-hexane. The eluate was then transferred to 1.5 mL amber sample vials, which were subsequently sealed and stored at a temperature of 4°C. This procedure was performed four times in total.

Volatile compounds were identified using a gas chromatograph-mass spectrometer (GC-MS, model 7890A-5975C; Agilent Technologies, California, USA) equipped with a DB-5 column. The temperature program was the same as that used in the GC-EAD analysis. Helium served as the carrier gas, with the GC-MS interface set to 250°C and the ion source temperature set to 230°C. The ionization current was set to 100 μA, the ionization energy to 70 eV, and the acceleration voltage to 6 kV. The scan range was between 50 and 350 m/z. Mass spectra data was compared to the NIST14 database, and the identities of the chemical components were confirmed by comparing retention times and mass spectra with authentic standards (Zhang et al., 2024).

### 2.5 Electroantennogram responses of individual winged *Aphis gossypii* to thirty-one volatile compounds

Active, robust winged *A. gossypii* were chosen for the experiment. The antennae of the test aphids were cleanly excised from the base with a surgical scalpel, and then mounted onto an EAG probe coated with Spectra360 conductive gel, using forceps. The instrument was calibrated to establish a stable baseline. We diluted the thirty-one identified volatiles using GC-MS with liquid paraffin, preparing five concentrations: 0.001, 0.01, 0.1, 1, and 10 μg/μL. A 10 μL sample of the solution for each volatile blend × concentration was applied to a 5 × 30 mm filter paper strip, which was then inserted into a 1 mL pipette tip from where EAG measurements were taken. Every stimulus lasted for 1 s, with a 30 s interval between consecutive stimulations. The sequence of stimulation was as follows: blank, control, five compound concentrations (from low to high), (*Z*)-3-hexen-1-ol, and control. Each compound or concentration was tested six times (Zhang et al., 2024). The positive control was liquid paraffin, (*Z*)-3-hexen-1-ol, utilized to assess the antennal activity.

### 2.6 Behavioral responses of winged *Aphis gossypii* to fourteen repellent compounds

#### 2.6.1 Y-tube olfactometer tests of individual compounds against winged aphids

Active, undamaged *A. gossypii* adults were selected and examined for their choice behavior toward individual compounds using a Y-tube olfactometer. Each arm of the Y-tube olfactometer was attached to a 1 mL centrifuge tube with the bottom removed. One tube included 20 μL of a slow-release formulation with a 1 μg/μL concentration of an active substance standard, while the other tube held liquid paraffin as a control. The airflow rate was set to 0.3 L/min. A solitary aphid was gently positioned at the base of the Y-tube olfactometer arm using a fine brush. The timing started when the aphid got halfway down the basal arm. Aphid behavior was gauged based on the following standards: If an aphid crossed a line one-third into either side arm and stayed there for over 5 s, it was considered to have made that choice. If no clear choice behavior was observed within 5 min, the aphid was recorded as a non-responder. After testing five aphids, the positions of the Y-tube olfactometer arms were swapped. A new clean Y-tube olfactometer replaced the previous one after examining ten aphids. After 10 min, the sample solution was replenished, and the airflow direction was reversed. Each treatment involved testing a total of 90 winged aphids, with each aphid being tested only once.

#### 2.6.2 Behavioral test of individual compounds against winged aphids in Petri dishes

A 15-cm diameter Petri dish was divided into four equal sections using a marking pen. Fresh cotton leaves were cut into 1 cm diameter discs. For the treatment group, 50 μL of a 1 μg/μL concentration of an active substance standard was applied to the leaf discs, whereas liquid paraffin was applied to the control group discs. The two types of discs were placed diagonally opposite each other inside the test cages. Subsequently, 30 aphids, possessing intact antennae and comparable in size and robustness to the other aphids in the experiment, were placed in the center of the Petri dishes. The dishes were then covered with an 80-mesh nylon gauze and placed in a 25°C artificial climate chamber with 60%–75% RH, a 16:8 L:D photoperiod, and 600 lx light intensity. After 30 min, the number of aphids in each of the four sections of the Petri dishes was recorded. Each treatment was repeated three times.

### 2.7 Data analysis

The Y-tube olfactometer bioassays and the individual compound repellency tests’ aphid choice data were analyzed using a Chi-square (*χ*²) test, with the null hypothesis assuming no preference between control and treatment arms. The number of aphids making a choice served as the response variable and those non-responding were excluded from the analysis. This method is frequently employed in Y-tube olfactometer studies for assessing olfactory preference (Zhang et al., 2024). In the cage choice experiment, we calculated the repellent index (RI) of the test plants based on the number of aphid selections and then we performed comparative analysis between the fifteen plants using one-way analysis of variance (ANOVA) followed by Tukey’s HSD test (*P* < 0.05) to determine significant differences. In the electroantennogram (EAG) experiment, following EAG recordings, the normalized EAG response values (V values) were subjected to one-way ANOVA and Tukey’s test to identify significant differences between test compounds and control stimuli across different concentrations. For the cage-choice behavioral test and the antennal potential response test of winged aphids, statistical analyses were executed using SPSS version 25.0 software (IBM Corporation, Armonk, NY, USA). Considering the data adheres to a normal distribution, differences between treatments were evaluated using one-way analysis of variance (ANOVA), followed by *post-hoc* comparisons with the LSD multiple comparison tests.

The repellency index (RI) was calculated as per Equation 1.


(1) $$\text{RI}=(\frac{\text{C}-\text{T}}{\text{C}+\text{T}})\times 100$$

Where C is the number of adults on control plants and T is the number of adults on treated plants (Pavela, 2011). The relative EAG response values were calculated as per Equation 2.


(2) V=CT−CK

Where V represents the absolute EAG value of *A. gossypii* in response to the stimulus, CT stands for the EAG amplitude of the tested stimulus, and CK signifies the average EAG amplitude of the control (liquid paraffin).

## 3 Results

### 3.1 Screening of fifteen plant species for repellent activity against winged *A. gossypii*

**Figure 1:**
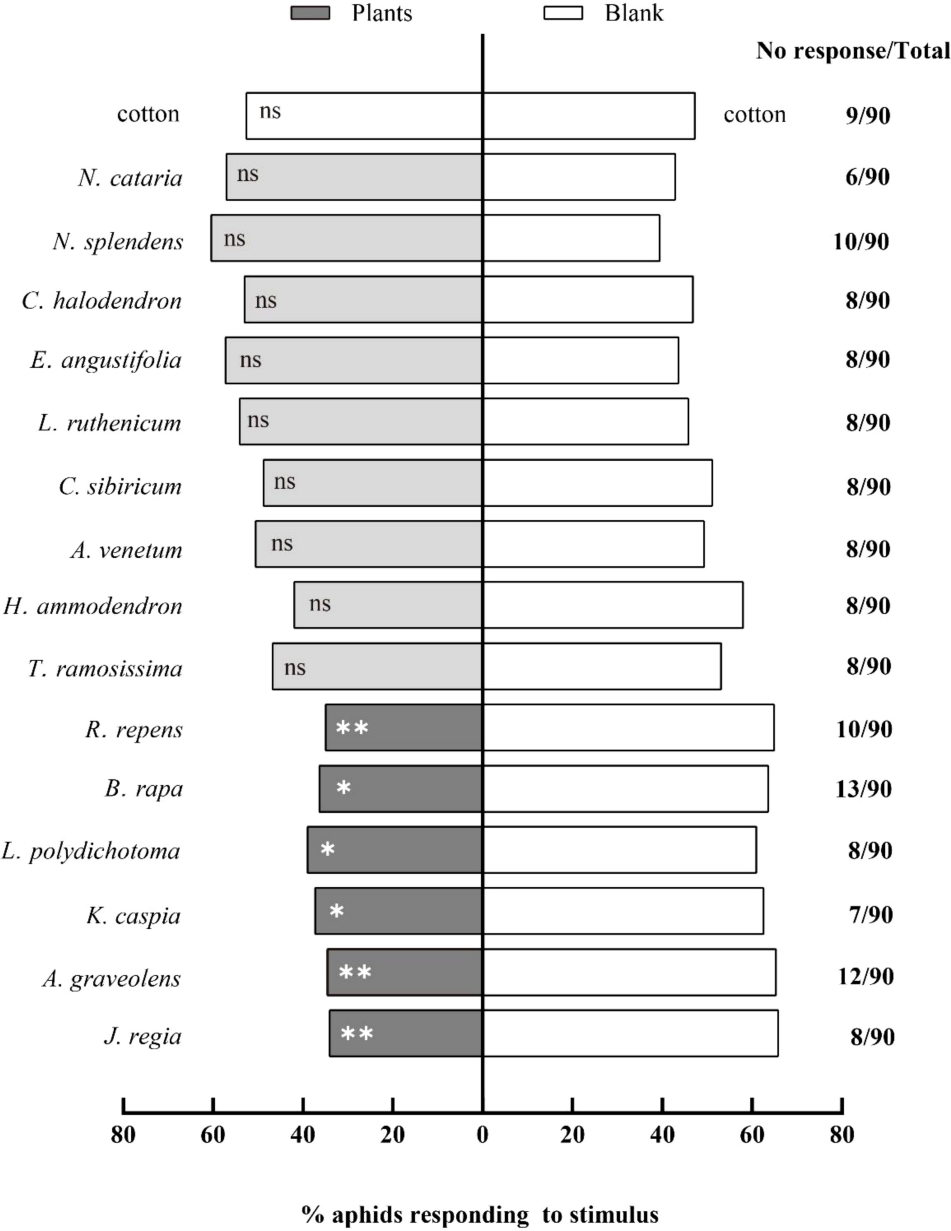
Behavioral responses of winged *Aphis gossypii* to different plants in Y-tube olfactometer assays. The control for this test was clean air. The *χ* ^2^ tests were used. Significant difference (ns: *P* > 0.05, *P < 0.05, **P < 0.01, ***P < 0.001).

A total of fifteen potentially repellent plant species were tested using a Y-tube olfactometer. Out of these fifteen species, six plants significantly repelled cotton aphids: *A. graveolens*, *J. regia*, *R. repens*, *K. caspia*, *L. polydichotoma*, and *B. rapa*. Winged *A. gossypii* showed a highly significant repellency response to the following three test plants (*P* < 0.01) (**Figure 1**): *A. graveolens* (*χ²* = 7.38, df = 1, *P* = 0.007), *J. regia* (*χ²* = 8.25, df = 1, *P* = 0.004), and *R. repens* (*χ²* = 7.20, df = 1, *P* = 0.007). Three other test plants demonstrated a lower, but still significant repellency response (*P* < 0.05): *K. caspia* (*χ²* = 5.31, df = 1, *P* = 0.020), *L. polydichotoma* (*χ²* = 3.95, df = 1, *P* = 0.046), and *B. rapa* (*χ²* = 5.73, df = 1, *P* = 0.017). However, no significant response was observed toward the volatiles of nine test plants (*P* > 0.05): *T. ramosissima*, *H. ammodendron*, *A. venetum*, *C. sibiricum*, *L. ruthenicum*, *E. angustifolia*, *N. cataria*, *N.* sp*lendens*, and *C. halodendron*.

**Figure 2:**
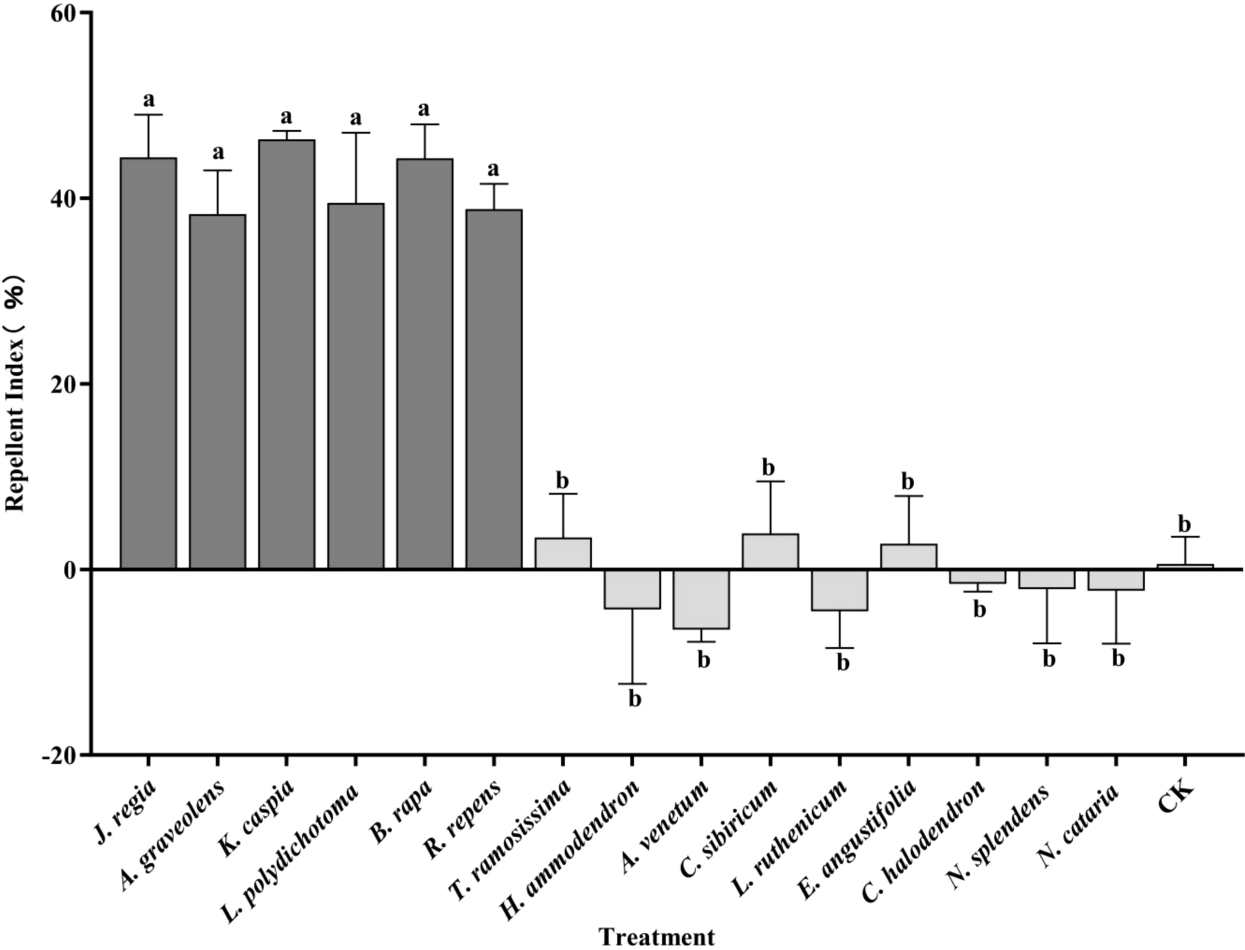
Host selection behavior of winged *Aphis gossypii* among different plant species at 24 h post-release. Different lowercase letters (a, b) indicate statistically significant differences (*P* < 0.05). The error bars indicate standard error (SE). Statistical comparisons were conducted using a one-way analysis of variance (ANOVA).

The RIs for the fifteen plant species that were tested are presented in **Figure 2**. Out of these, six species exhibited significant repellency to winged *A. gossypii*. These were: *K. caspia* (*RI* = 46.35), *J. regia* (*RI* = 44.39), *B. rapa* (*RI* = 44.32), *L. polydichotoma* (*RI* = 39.51), *R. repens* (*RI* = 38.82), and *A. graveolens* (*RI* = 38.29). A significant difference was observed in the repellency indices between these six repellent plants (as a group) and the remaining nine plants (*P* < 0.05).

### 3.2 Collection and identification of volatile compounds from six repellent plant species

**Figure 3:**
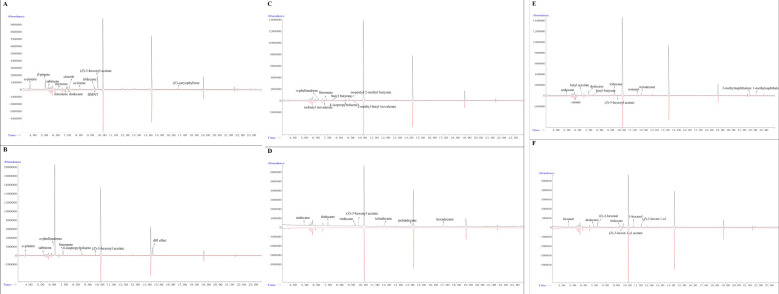
GC-MS analysis of volatile compounds from six plant species. **(A)** *J*. *regia*, **(B)** *A*. *graveolens*, **(C)** *K*. *caspia*, **(D)** *L. polydichotoma*, **(E)** *B*. *rapa*, and **(F)** *R. repens*.

GC-MS was used to analyze the volatile compounds released into the headspace of six previously collected plant species known for their repellent effects on winged *A. gossypii* (**Figure 3**). The results showed that *J. regia* volatiles exhibited the highest levels of *α*-pinene, *β*-pinene, and ocimene, while *A. graveolens* volatiles contained the highest level of *α*-phellandrene. *K. caspia* volatiles had the highest levels of *α*-phellandrene and isobutyl isovalerate, whereas *L. polydichotoma* volatiles had the highest level of pentadecane. Additionally, *B. rapa* volatiles possessed the highest level of alkanes, with *R. repens* volatiles showing the highest level of (*E*)-2-hexen-1-al (**Supplementary Table S2**).

### 3.3 Electroantennogram responses of individual winged *A. gossypii* to thirty-one volatile compounds

The antennal responses of winged *A. gossypii* to thirty-one compounds, as identified by GC-MS from headspace volatile collections, were tested. Out of these thirty-one compounds, sixteen induced significant EAG responses. Significant differences were noted in the responses of winged *A. gossypii* antennae to varying doses of sixteen compounds (*P* < 0.05). These include (1) eucalyptol, (2) (*E*)-2-hexenal, (3) 1-hexanol, (4) limonene, (5) myrcene, (6) *α*-pinene, (7) hexanal, (8) nonanal, (9) ocimene, (10) 3-carene, (11) isobutyl isovalerate, (12) n-butyl butanoate, (13) (*Z*)-3-hexen-1-yl acetate, (14) 2-methyl butyl isovalerate, (15) DMNT, and (16) (*Z*)-3-hexen-1-ol (**Table 2**). The remaining fifteen compounds, including *α*-phellandrene, 4-isopropyltoluene, 1-methylnaphthalene, 2-methylnaphthalene, (*E*)-caryophyllene, *β*-pinene, n-undecane, dodecane, tridecane, tetradecane, pentadecane, hexadecane, n-butyl acrylate, isopentyl 2-methylbutyrate, and sabinene, did not induce any significant EAG response at any of the tested concentrations (*P* > 0.05).

**Table 2:** Electroantennographic responses of winged *Aphis gossypii* to plant volatile compounds.

Compounds	0.001(μg/μl)	0.01(μg/μl)	0.1(μg/μl)	1(μg/μl)	10(μg/μl)
*α*-phellandrene	0.0033 ± 0.0033a	0.0067 ± 0.0067a	0.0133 ± 0.0033a	0.0133 ± 0.0033a	0.0200 ± 0.0075a
4-isopropyltoluene	0.0043 ± 0.0043a	0.0067 ± 0.0041a	0.0077 ± 0.0077a	0.0077 ± 0.0077a	0.0197 ± 0.0168a
eucalyptol	0.0300 ± 0.0058b	0.0300 ± 0.0058b	0.0267 ± 0.0033b	0.0333 ± 0.0067b	0.3500 ± 0.036a
1-methylnaphthalene	0.0042 ± 0.0042a	0.0075 ± 0.0075a	0.0092 ± 0.0022a	0.0225 ± 0.0113a	0.0092 ± 0.0022a
2-methylnaphthalene	0.0433 ± 0.0067a	0.0533 ± 0.0145a	0.0567 ± 0.0219a	0.0467 ± 0.0033a	0.0667 ± 0.0120a
nonanal	0.267 ± 0.0120ab	0.0100 ± 0.0100b	0.0200 ± 0.0058ab	0.0467 ± 0.0133a	0.0467 ± 0.0088a
(*E*)-2-hexen-1-al	0.0200 ± 0.0100c	0.0733 ± 0.0088bc	0.1567 ± 0.0240b	0.1633 ± 0.0145b	0.7567 ± 0.0633a
1-hexanol	0.0533 ± 0.0291d	0.0983 ± 0.0130cd	0.1517 ± 0.0117c	0.6617 ± 0.0319b	1.3217 ± 0.0263a
(*Z*)-3-hexen-1-ol	0.0233 ± 0.0033b	0.0433 ± 0.0120b	0.0433 ± 0.0233b	0.0600 ± 0.0208b	0.5200 ± 0.0569a
limonene	0.0137 ± 0.0068b	0.0167 ± 0.0167b	0.0367 ± 0.0088b	0.0300 ± 0.0058b	0.1567 ± 0.023a
(*E*)-caryophyllene	0.0100 ± 0.0100a	0.0167 ± 0.0120a	0.0133 ± 0.0133a	0.0267 ± 0.0176a	0.0433 ± 0.0067a
*β*-pinene	0.0600 ± 0.0288a	0.0967 ± 0.0296a	0.1067 ± 0.0203a	0.1267 ± 0.0088a	0.1100 ± 0.0208a
myrcene	0.0233 ± 0.0067b	0.0267 ± 0.0133b	0.0233 ± 0.0067b	0.0200 ± 0.0058b	0.0967 ± 0.0120a
*α*-pinene	0.0910 ± 0.0410b	0.1010 ± 0.0380b	0.0910 ± 0.0364b	0.2110 ± 0.0309a	0.2477 ± 0.0114a
n-undecane	0.0100 ± 0.0058a	0.0267 ± 0.0145a	0.0300 ± 0.0033a	0.0467 ± 0.0203a	0.0300 ± 0.0116a
dodecane	0.0200 ± 0.0116a	0.0767 ± 0.0186a	0.0900 ± 0.0208a	0.0600 ± 0.0058a	0.0500 ± 0.0153a
tridecane	0.0133 ± 0.0033a	0.0067 ± 0.0033a	0.0033 ± 0.0033a	0.0033 ± 0.0033a	0.0067 ± 0.0067a
tetradecane	0 ± 0a	0.0033 ± 0.0033a	0 ± 0a	0.0033 ± 0.0033a	0.0033 ± 0.0033a
pentadecane	0.0100 ± 0a	0.0100 ± 0.0058a	0.0100 ± 0.0058a	0.0100 ± 0.0058a	0.0067 ± 0.0033a
hexadecane	0.0100 ± 0.0058a	0.0067 ± 0.0067a	0.0167 ± 0.0120a	0.0167 ± 0.0088a	0.0100 ± 0.0058a
ocimene	0.0167 ± 0.0088b	0.0137 ± 0.0088b	0.0037 ± 0.0032b	0.0100 ± 0.0100b	0.0713 ± 0.0149a
3-carene	0.0400 ± 0.0058b	0.0367 ± 0.0267b	0.0533 ± 0.0176b	0.0833 ± 0.0145b	0.1833 ± 0.0145a
isobutyl isovalerate	0.0400 ± 0.0173c	0.0500 ± 0.0252c	0.0733 ± 0.0120bc	0.1333 ± 0.0176ab	0.1433 ± 0.0233a
butyl butyrate	0.0100 ± 0.0058d	0.0167 ± 0.0088cd	0.0367 ± 0.0033c	0.0733 ± 0.0033b	0.1833 ± 0.0088a
hexanal	0.0100 ± 0.0100c	0.0100 ± 0c	0.0200 ± 0.0058c	0.2600 ± 0.0100b	0.5367 ± 0.0167a
(*Z*)-3-hexenyl acetate	0.0600 ± 0.0116c	0.0900 ± 0.0153c	0.0967 ± 0.0033c	0.2133 ± 0.0120b	0.7433 ± 0.0463a
n-butyl acrylate	0.0133 ± 0.0088a	0.0233 ± 0.0088a	0.0167 ± 0.0067a	0.0200 ± 0.0058a	0.0200 ± 0.0058a
2-methylbutyl Isovalerate	0.0117 ± 0.0041c	0.0103 ± 0.0012c	0.0070 ± 0.0025c	0.0733 ± 0.0033b	0.1467 ± 0.0240a
isopentyl 2-methylbutyrate	0.0200 ± 0.0058a	0.0067 ± 0.0033a	0.0167 ± 0.0088a	0.0067 ± 0.0067a	0.0167 ± 0.0120a
DMNT	0.0100 ± 0.0058d	0.0167 ± 0.0088cd	0.0367 ± 0.0033c	0.0733 ± 0.0033b	0.1833 ± 0.0088a
sabinene	0.0100 ± 0.0058a	0.0100 ± 0.0058a	0.0100 ± 0.0058a	0.0200 ± 0.0033a	0.0233 ± 0.0058a

Statistical comparisons were conducted using a one-way analysis of variance (ANOVA). Different lowercase letters (a,b,c,d) indicate statistically significant differences (*P* < 0.05).

### 3.4 Behavioral tests of fourteen repellent compounds against winged *A. gossypii*

**Figure 4:**
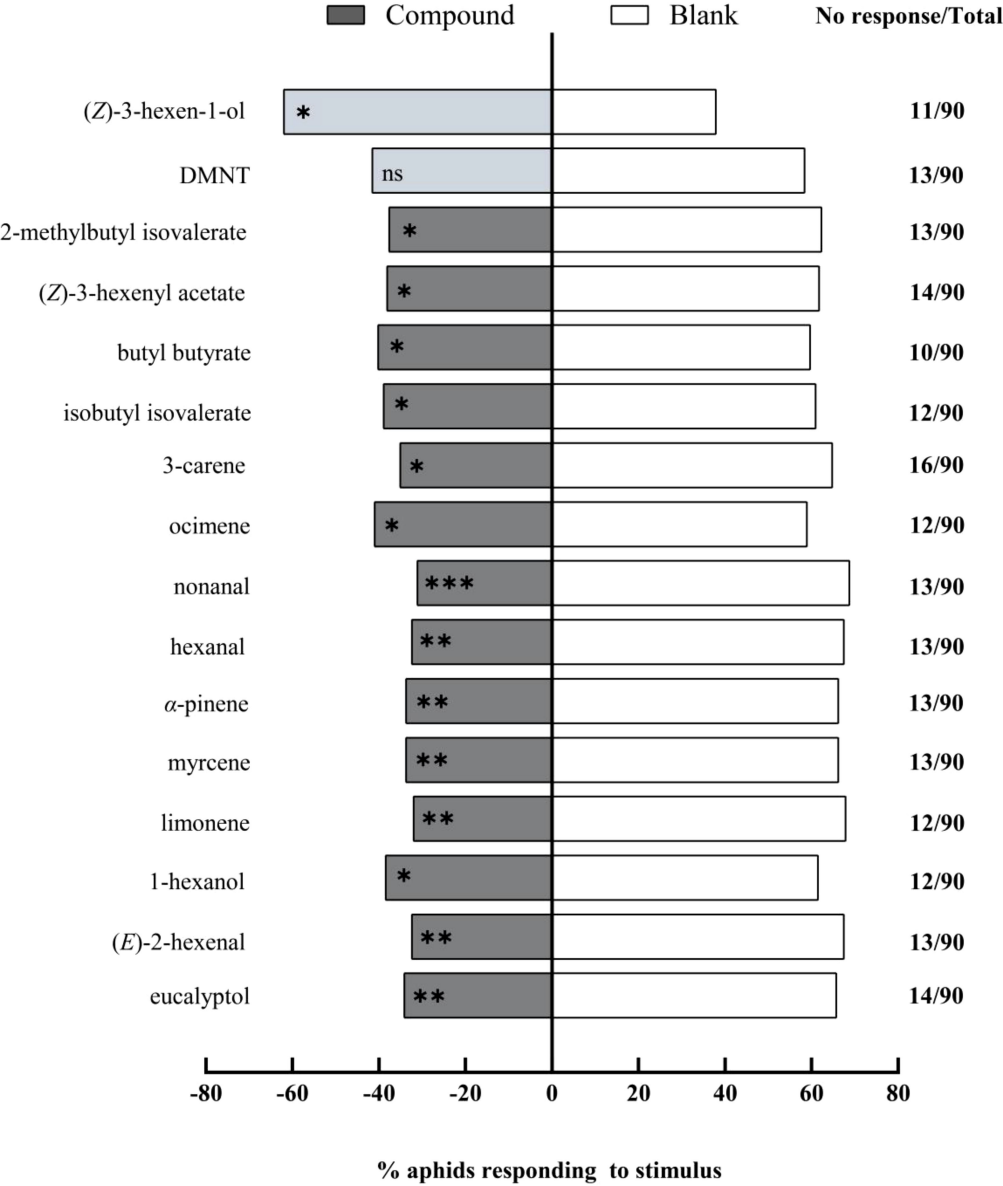
Behavioral responses of winged *Aphis gossypii* to sixteen volatile compounds (1 μg/μL) in Y-tube olfactometer assays. The control for this test was liquid paraffin. The *χ* ^2^ tests were used. Significant difference (ns: *P* > 0.05, *P < 0.05, **P < 0.01, ***P < 0.001).

Y-tube olfactory preference assays were conducted to evaluate sixteen chemical compounds. These elicited significant electrophysiological responses in *A. gossypii* antennae, with each at a concentration of 1 μg/μL. Fourteen compounds were found to be repellent (**Figure 4**). Among these, nonanal (*χ²* = 10.92, df = 1, *P* = 0.001) had an extremely significant repellent effect, while eucalyptol (*χ²* = 7.58, df = 1, *P* = 0.006), (*E*)-2-hexenal (*χ²* = 9.47, df = 1, *P* = 0.002), limonene (*χ²* = 10.05, df = 1, *P* = 0.002), myrcene (*χ²* = 8.12, df = 1, *P* = 0.004), *α*-pinene (*χ²* = 8.12, df = 1, *P* = 0.004), and hexanal (*χ²* = 9.47, df = 1, *P* = 0.002) exhibited very significant repellent effects. Additionally, 1-hexanol (*χ²* = 4.15, df = 1, *P* = 0.042), ocimene (*χ²* = 4.15, df = 1, *P* = 0.042), 3-carene (*χ²* = 6.54, df = 1, *P* = 0.011), isobutyl isovalerate (*χ²* = 4.15, df = 1, *P* = 0.042), n-butyl butanoate (*χ²* = 4.05, df = 1, *P* = 0.044), (*Z*)-3-hexen-1-yl acetate (*χ²* = 4.26, df = 1, *P* = 0.039), and 2-methyl butyl isovalerate (*χ²* = 4.69, df = 1, *P* = 0.031) prevailed as significantly repellent. In contrast, (*Z*)-3-hexen-1-ol (*χ²* = 4.57, df = 1, *P* = 0.033) exhibited significant attraction for the aphids. Yet, DMNT (*χ²* = 2.20, df = 1, *P* = 0.140) did not significantly alter the aphids’ choice behavior.

**Figure 5:**
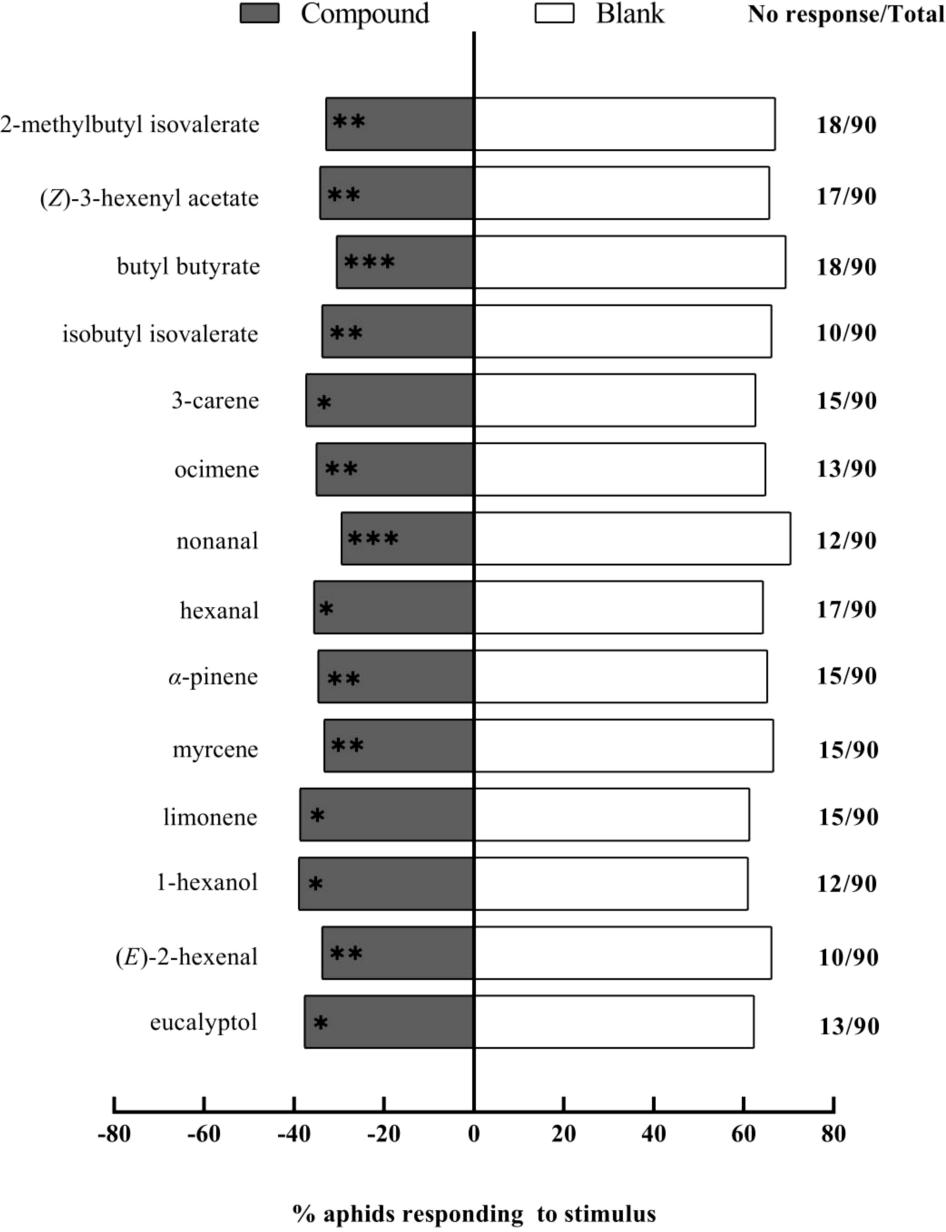
Repellent effects of fourteen chemical compounds (1 μg/μL) against winged *Aphis gossypi*i in Petri dish assays. The control for this test was liquid paraffin. The *χ* ^2^ tests were used. Significant difference (ns: *P* > 0.05, *P < 0.05, **P < 0.01, ***P < 0.001).

In the Petri dish choice assay, we tested the fourteen compounds previously screened by the Y-tube olfactometer, each at a concentration of 1 μg/μL, on winged *A. gossypii*. Our findings indicate that nonanal (*χ²* = 13.13, df = 1, *P* < 0.001) and butyl butyrate (*χ²* = 10.89, df = 1, *P* < 0.001) had an extremely significant repellent effect (**Figure 5**). (*E*)-2-hexenal (*χ²* = 8.45, df = 1, *P* = 0.004), *α*-pinene (*χ²* = 7.05, df = 1, *P* = 0.008), ocimene (*χ²* = 6.87, df = 1, *P* = 0.009), isobutyl isovalerate (*χ²* = 8.45, df = 1, *P* = 0.004), 2-methyl butyl isovalerate (*χ²* = 9.56, df = 1, *P* = 0.002), myrcene (*χ²* = 8.33, df = 1, *P* = 0.004), and (*Z*)-3-hexenyl acetate (*χ²* = 7.25, df = 1, *P* = 0.007) all had very significant repellent effects. Furthermore, 1-hexanol (*χ²* = 4.15, df = 1, *P* = 0.042), eucalyptol (*χ²* = 4.69, df = 1, *P* = 0.030), 3-carene (*χ²* = 4.81, df = 1, *P* = 0.028), limonene (*χ²* = 3.85, df = 1, *P* = 0.050), and hexanal (*χ²* = 6.04, df = 1, *P* = 0.014) exhibited significant repellent effects on the aphids.

## 4 Discussion

Plant volatile compounds, renowned for their natural pest-repelling properties, have attracted increasing attention, especially in the management of economically significant pests such as *A. gossypii* (War et al., 2011). In this study, we identified local plants from Xinjiang that demonstrated repellent effects on winged *A. gossypii*, and their responsible volatile compounds. Initially, we conducted behavioral assays, featuring Y-tube olfactometer and cage-choice tests, in fifteen species of Xinjiang plants. Six plants – *A. graveolens*, *J. regia*, *R. repens*, *K. caspia*, *L. polydichotoma*, and *B. rapa* –proved to significantly repel winged *A. gossypii*. We employed a headspace collection method to capture volatile compounds from these plants, and GC-MS analysis identified thirty-one distinct volatile compounds released by the test plants. Subsequently, we performed electroantennogram (EAG) recordings to gauge antennal responses of winged *A. gossypii* to these thirty-one volatile compounds. EAG assays disclosed that sixteen compounds triggered significant antennal responses from winged *A. gossypii*. Lastly, further verification through Y-tube olfactometer and Petri dish experiments identified fourteen compounds – including (1) n-butyl butanoate, (2) eucalyptol, (3) (*E*)-2-hexenal, (4) limonene, (5) *α*-pinene, (6) hexanal, (7) nonanal, (8) ocimene, (9) isobutyl isovalerate, (10) (*Z*)-3-hexen-1-yl acetate, (11) 1-hexanol, (12) myrcene, (13) 3-carene, and (14) 2-methyl butyl isovalerate – which showed significant repellent effects against *A. gossypii*. These findings offer fresh theoretical support for the utilization of plant volatiles in the environmentally friendly control of *A. gossypii*.

**Figure 6:**
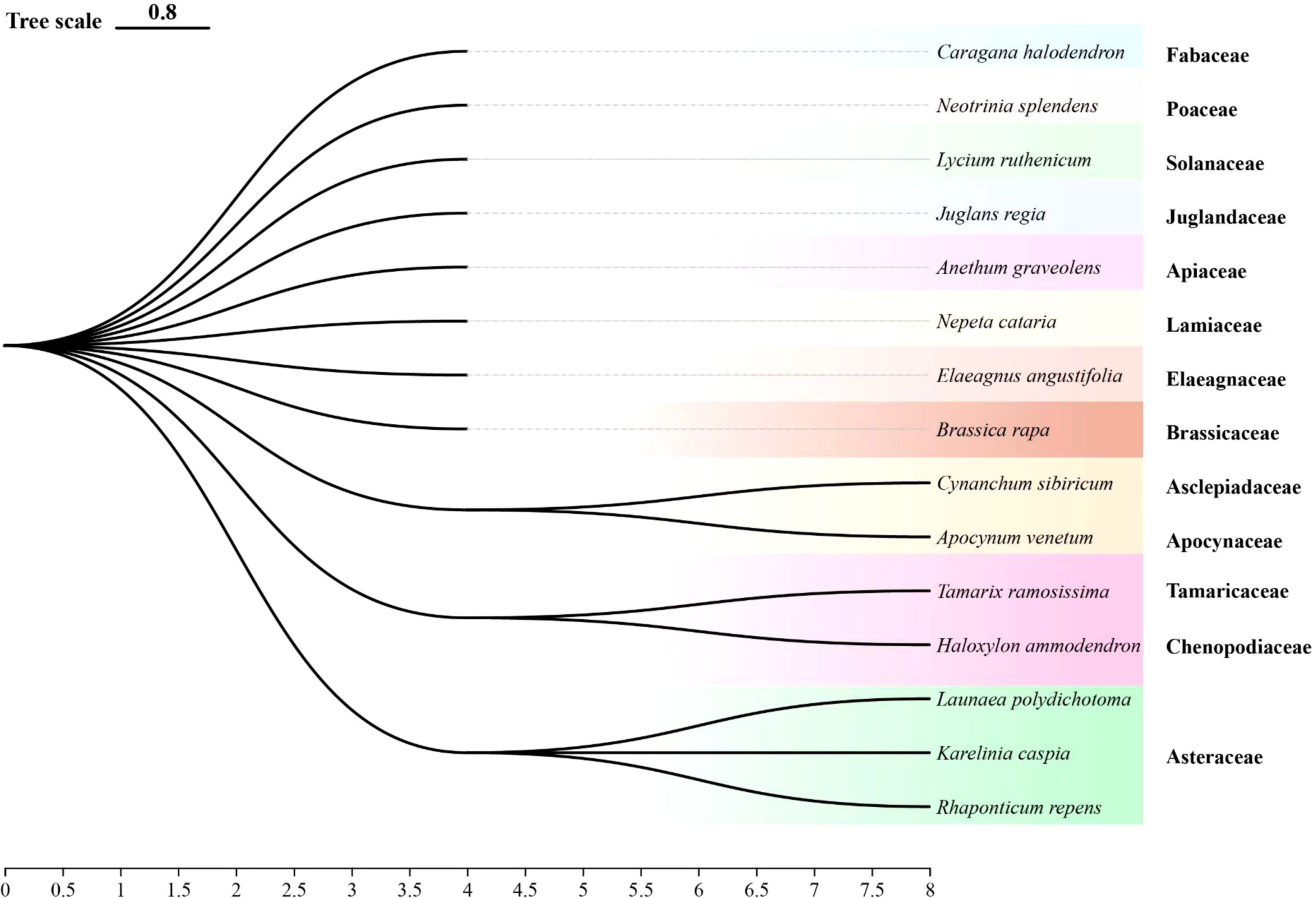
Phylogenetic tree of test plants. The phylogenetic tree visually illustrates the evolutionary relationships among various plant species.

Many plants naturally emit distinct volatile compounds that influence the host-plant finding behavior of herbivorous insects (Finch and Collier, 2012). This study found that *A. graveolens*, *J. regia*, *R. repens*, *K. caspia*, *L. polydichotoma* and *B. rapa.* significantly repelled *A. gossypii*. Prior work demonstrated that the intercropping of tomatoes with *A. graveolens* noticeably repelled the whitefly *Bemisia tabaci* Gennadius (Padala et al., 2023) and that *J. regia* volatiles repel *Cydia pomonella* L (Li et al., 2024). These findings align with the repellent effects observed for *A. graveolens* and *J. regia* in this study. While no previous studies have specifically reported the repellent effects of *R. repens*, *K. caspia*, or *L. polydichotoma* volatiles, it is noteworthy that these plants belong to the *Asteraceae* family (**Figure 6**), which consists of several species recognized for their ability to repel herbivorous insects. For example, *Tagetes patula* L. has been shown to repel three species of Tortricidae (Lepidoptera: Tortricidae) effectively in an Apple Orchard (Song et al., 2014), and various species of *Artemisia* exhibit strong repellent effects against aphids (Yang et al., 2024). While research on the effects of turnip (*B. rapa*) volatiles on insect behavior is limited, key volatiles in *B. rapa*, including myrcene, limonene, ocimene, and *α*-pinene, are known to repel certain herbivorous insects (Kugimiya et al., 2010). Consequently, the repellent effects of *B. rapa* can be attributed to its volatile compounds.

GC-MS is an effective technique for the qualitative and quantitative analysis of volatile compounds emitted from plants (Wang et al., 2018). In this study, GC-MS was used to analyze volatiles from six plant species, identifying a variety of volatile compounds. The compounds align with those reported in other studies, reaffirming the diversity and reproducibility of plant volatile profiles. For instance, volatiles from *J. regia* reported in the literature include *α*-pinene, *β*-pinene, myrcene, ocimene, sabinene, limonene, tridecane, and (*E*)-caryophyllene (Farag, 2008). However, dodecane, eucalyptol, and DMNT were not detected in some research, possibly due to differences in walnut species, growth stages, or environmental conditions of the sampled plants (Farag, 2008; Okatan et al., 2022). This highlights that the diversity of volatile compounds in plants is not only species-dependent but also influenced by environmental factors and growth conditions. In the current study, the compounds detected in the volatiles of *A. graveolens* closely matched those identified by Ramadan et al. (2013), who used steam distillation to collect the plant’s volatiles. The results of this study are in agreement with Ramadan’s findings, but some differences in other compounds were also observed. These discrepancies could be attributed to variations in volatile collection methods, experimental conditions, or sample sources (Ramadan et al., 2013). Many volatile compounds exhibit biological activity, influencing insect behavior, regulating plant interactions, and providing resistance to pests and diseases (Bruce and Pickett, 2011). Thus, the volatile compounds identified in this study provide valuable insights for insect behavioral research and establish a theoretical foundation for the potential application of natural plant volatiles in pest management.

Insect antennae contain olfactory receptors that play a crucial role in detecting volatile chemical signals. These receptors bind to external volatile molecules, triggering neural signal transmission and prompting the insect to exhibit behavioral responses, such as approaching or avoiding specific volatile sources (Hallem et al., 2006). EAG technology facilitates real-time, quantitative measurement of antennal receptor responses to specific volatile molecules and is considered one of the standard methods for studying insect olfactory behavior (Ngumbi et al., 2010; Liu et al., 2024). In this study, we tested the antennal responses of winged *A. gossypii* to thirty-one compounds. Of those, sixteen compounds induced significant EAG responses. Previous research has shown that different insect species present distinct EAG response curves to the same compounds (Ngumbi et al., 2010). For instance, some studies have confirmed that *α*-pinene, *β*-pinene, and eucalyptol do not elicit antennal responses in *C. pomonella* (Knudsen et al., 2017). However, other studies have shown that these same compounds can induce antennal responses in the crambid moth *Conogethes punctiferalis* Guenée (Du et al., 2016). Similarly, our results demonstrate that ocimene can elicit significant antennal responses in *A. gossypii.* In contrast, ocimene did not induce antennal responses in the tachinid fly *Blepharipa zebina* Walker (Xu et al., 2007). These discrepancies are likely attributable to differences in insect species and their ecology. Other studies have confirmed that different herbivorous insects may exhibit varying behavioral responses to the same compounds (Park et al., 2002). For instance, ocimene and eucalyptol repel the aphid *Dysaphis plantaginea* Passerini (Dieudonné et al., 2022). Similarly, *α*-pinene and limonene exhibit repellent effects on *Macdunnoughia crassisigna* Warren (Wang et al., 2024b); this aligns with our findings. In contrast, 1-hexanol attracted the aphid *Sitobion avenae* Fabricius significantly (Mitra et al., 2021). Likewise, (*E*)-2-hexenal was attractive to the aphid *Acyrthosiphon pisum* Harris (Huang et al., 2022), both of which contrast with our findings on the reactions of different aphids to the same compounds. These discrepancies may arise from variations in experimental subjects (Park et al., 2002). Additionally, behavioral responses are often dose-dependent, and different concentrations of compounds can result in different experimental outcomes (Yang et al., 2016; Dieudonné et al., 2022).

By identifying and screening plant volatiles with significant repellent effects, this research offers potential innovative strategies for future management of *A. gossypii*. The plant-derived repellent compounds identified in this study could be implemented in agricultural settings through several practical approaches. First, the development of slow-release formulations containing key repellent compounds such as nonanal, eucalyptol, and *α*-pinene could provide sustained protection in cotton fields, like the successful application of essential oil nanoformulations against tomato borer (Campolo et al., 2017). These formulations could be deployed as dispensers placed strategically throughout fields, creating a repellent barrier against incoming winged aphids. Second, the identified repellent plants, particularly *R. repens*, *K. caspia*, and *L. polydichotoma*, could be incorporated into push-pull strategies, where these plants are grown around cotton field boundaries to repel aphids while simultaneously using attractive plants elsewhere to trap them, as demonstrated in successful aphid management systems (Cook et al., 2007; Yang et al., 2023). Such strategies have the advantage of reducing reliance on chemical pesticides, minimizing environmental contamination, and lessening the impact of chemical agents on non-target organisms. Future research should focus on the practical implementation of these plant volatiles in agricultural settings, evaluating their effects on *A. gossypii* population dynamics, and monitoring their persistence in the field to assess their efficacy duration.

## 5 Conclusion

The identification of plant volatiles with repellent effects against *A. gossypii* provides a promising foundation for developing sustainable, environmentally friendly pest management strategies in cotton production. Our comprehensive screening of fifteen plant species revealed that *A. graveolens*, *K. caspia*, *J. regia*, *L. polydichotoma*, *B. rapa*, and *R. repens* significantly repel *A. gossypii*, representing valuable intercropping candidates for cotton fields. The fourteen repellent volatile compounds we identified, including eucalyptol, (*E*)-2-hexenal, 1-hexanol, limonene, myrcene, *α*-pinene, hexanal, nonanal, ocimene, 3-carene, isobutyl isovalerate, n-butyl butanoate, (*Z*)-3-hexenyl acetate, and 2-methyl butyl isovalerate, could be formulated into botanical repellents or slow-release dispensers for field deployment. These natural compounds offer an alternative to conventional pesticides, potentially reducing chemical inputs in cotton and preserving beneficial insects within integrated pest management systems.

## Data Availability

The raw data supporting the conclusions of this article are available from the corresponding author upon reasonable request.

## Supplementary material

Supplementary Table 1 Standardized information on substances.

Supplementary Table 2 Quantitative composition of volatile compounds in six plant species (mg/compound).
