# Supplementary material for: Exploring repellency of odors from non-host plants native to Xinjiang, China to Aphis gossypii
Source: Front Plant Sci. 2025 May 28;16:1563752. doi: 10.3389/fpls.2025.1563752 (PMC12153815; doi:10.3389/fpls.2025.1563752)
Supplement: Supplementary Table 1 — Standardized information on substances. [file Table1.docx]

**Table S1.** Standardized information on substances

| **Standard compounds** | **Retention time (min)** | **CAS No.** | **Purity (%)** |
| --- | --- | --- | --- |
| *α*-phellandrene | 6.191 | 4221-98-1 | 65 |
| 4-isopropyltoluene | 8.506 | 99-87-6 | 98 |
| eucalyptol | 7.162 | 470-82-6 | 99 |
| 1-methylnaphthalene | 22.258 | 90-12-0 | 96 |
| 2-methylnaphthalene | 21.733 | 91-57-6 | 97 |
| nonanal | 11.515 | 124-19-6 | 96 |
| (*E*)-2-hexen-1-al | 7.275 | 6728-26-3 | 98 |
| 1-hexanol | 10.552 | 111-27-3 | 99 |
| (*Z*)-3-hexen-1-ol | 11.294 | 928-96-1 | 98 |
| limonene | 6.898 | 5989-54-8 | 95 |
| (*E*)-caryophyllene | 16.604 | 87-44-5 | 98 |
| *β*-pinene | 5.085 | 18172-67-3 | 98 |
| myrcene | 6.139 | 123-35-3 | 90 |
| *α*-pinene | 3.737 | 80-56-8 | 98 |
| n-undecane | 4.955 | 1120-21-4 | 99.8 |
| dodecane | 6.989 | 112-40-3 | 98 |
| tridecane | 9.338 | 629-50-5 | 99 |
| tetradecane | 11.801 | 629-59-4 | 99.5 |
| pentadecane | 14.255 | 629-62-9 | 99.8 |
| hexadecane | 16.873 | 544-76-3 | 99.5 |
| ocimene | 8.085 | 13877-91-3 | 90 |
| 3-carene | 5.814 | 13466-78-9 | 90 |
| isobutyl isovalerate | 6.741 | 589-59-3 | 98 |
| butyl butyrate | 7.34 | 109-21-7 | 99.5 |
| hexanal | 4.595 | 66-25-1 | 97 |
| (*Z*)-3-hexenyl acetate | 9.629 | 3681-71-8 | 98 |
| n-butyl acrylate | 6.386 | 141-32-2 | 99.5 |
| 2-methyl butyl isovalerate | 9.165 | 2445-77-4 | 98 |
| isopentyl 2-methyl butyrate | 8.801 | 27625-35-0 | 98 |
| DMNT | 9.416 | 19945-61-0 | 96 |
| sabinene | 5.31 | 3387-41-5 | 70 |
