# Supplementary material for: Exploring repellency of odors from non-host plants native to Xinjiang, China to Aphis gossypii
Source: Front Plant Sci. 2025 May 28;16:1563752. doi: 10.3389/fpls.2025.1563752 (PMC12153815; doi:10.3389/fpls.2025.1563752)
Supplement: Supplementary Table 2 — Quantitative composition of volatile compounds in six plant species (mg/compound). [file Table2.docx]

**Table S2.** Quantitative composition of volatile compounds in six plant species (mg/compound)

|  | ***J. regia*** | ***A. graveolens*** | ***K. caspia*** | ***L. polydichotoma*** | ***B. rapa*** | ***R. repens*** |
| --- | --- | --- | --- | --- | --- | --- |
| *α*-phellandrene | — | 1979.14 | 21.85 | — | — | — |
| 4-isopropyltoluene | — | 2.76 | 7.89 | — | — | — |
| eucalyptol | 68.36 | — | — | — | — | — |
| 1-methylnaphthalene | — | — | — | — | 1.87 | — |
| 2-methylnaphthalene | — | — | — | — | 1.04 | — |
| nonanal | — | — | — | — | 7.54 | — |
| (*E*)-2-hexen-1-al | — | — | — | — | — | 79.22 |
| 1-hexanol | — | — | — | — | — | 16.28 |
| (*Z*)-3-hexen-1-ol | — | — | — | — | — | 14.08 |
| limonene | 92.72 | 89.77 | 6.74 | — | — | — |
| (*E*)-caryophyllene | 33.62 | — | — | — | — | — |
| *β*-pinene | 242.30 | — | — | — | — | — |
| myrcene | 43.99 | — | — | — | — | — |
| *α*-pinene | 141.73 | 37.31 | — | — | — | — |
| n-undecane | — | — | — | 2.85 | 1.64 | — |
| dodecane | 7.66 | — | — | 47.48 | 56.29 | 8.49 |
| tridecane | 20.67 | — | — | 61.86 | 80.03 | 23.49 |
| tetradecane | — | — | — | 40.03 | 26.53 | — |
| pentadecane | — | — | — | 290.98 | — | — |
| hexadecane | — | — | — | 13.96 | — | — |
| ocimene | 128.76 | — | — | — | — | — |
| 3-carene | — | — | — | — | 2.08 | — |
| isobutyl isovalerate | — | — | 19.76 | — | — | — |
| butyl butyrate | — | — | 7.18 | — | 12.20 | — |
| hexanal | — | — | — | — | — | 9.26 |
| (*Z*)-3-hexenyl acetate | 55.98 | 1.72 | — | 8.07 | 3.87 | 12.51 |
| n-butyl acrylate | — | — | — | — | 6.25 | — |
| 2-methyl butyl isovalerate | — | — | 8.21 | — | — | — |
| isopentyl 2-methyl butyrate | — | — | 7.7 | — | — | — |
| DMNT | 6.99 | — | — | — | — | — |
| sabinene | 19.27 | 3.67 |  |  |  |  |

Note: The numbers in the table are the amounts of the compounds per 100 g of the test plant’s volatiles, based on 4 h of head space capture of volatiles.
